# OMNI-CONV: Generalization of the Omnidirectional Distortion-Aware Convolutions

**DOI:** 10.3390/jimaging9020029

**Published:** 2023-01-28

**Authors:** Charles-Olivier Artizzu, Guillaume Allibert, Cédric Demonceaux

**Affiliations:** 1 Université Côte d’Azur, CNRS, I3S, 06900 Sophia Antipolis, France; 2 ImViA, Université Bourgogne Franche-Comté, 21000 Dijon, France

**Keywords:** equirectangular images, distortion-aware convolution, computer vision

## Abstract

Omnidirectional images have drawn great research attention recently thanks to their great potential and performance in various computer vision tasks. However, processing such a type of image requires an adaptation to take into account spherical distortions. Therefore, it is not trivial to directly extend the conventional convolutional neural networks on omnidirectional images because CNNs were initially developed for perspective images. In this paper, we present a general method to adapt perspective convolutional networks to equirectangular images, forming a novel distortion-aware convolution. Our proposed solution can be regarded as a replacement for the existing convolutional network without requiring any additional training cost. To verify the generalization of our method, we conduct an analysis on three basic vision tasks, i.e., semantic segmentation, optical flow, and monocular depth. The experiments on both virtual and real outdoor scenarios show our adapted spherical models consistently outperform their counterparts.

## 1. Introduction

Omnidirectional optical cameras can effectively capture their environment in a single shot thanks to their ultra-wide field of view (FoV). As a result, many robotic applications are interested in using such a type of image that can provide rich information about the scene, especially helpful for obstacle avoidance. Various recent works have shown the great potential of omnidirectional images, such as [1,2] for simultaneous visual localization and mapping (SLAM) and, more recently, ref. [3] on deep reinforcement learning (DRL). These solutions have shown better performances than their counterparts based on conventional images with a limited FoV.

Recent learning-based methods have greatly advanced the research for various vision and robotic tasks. This can be mainly contributed to the fast development of a GPU but more importantly to a large number of labeled datasets. Nevertheless, most existing datasets are with perspective images, with few datasets collected by omnidirectional sensors. Indeed, building an accurate and complete dataset is labor intensive and time consuming. In addition, omnidirectional sensors capable of extracting the ground truth are rare, complex to calibrate, and often subject to reconstruction errors. There are several recent attempts to build benchmark spherical datasets, such as Matterport3D [4] and Standford-2D3D [5]. However, these works were built virtually and with indoor scenes. Even though we can train networks on these datasets, extending the application to real cases or outdoor scenes is not trivial. Hence, developing a novel method to adapt from the networks pretrained on perspective images is highly demanded for omnidirectional applications.

As suggested in [6], all spherical projections come with distortions. In particular, equirectangular images, commonly used for their easy readability and classical rectangular format, suffer from significant distortions in the polar regions. Because of this non-linearity, objects appear differently at different latitudes. To tackle this issue, several approaches propose to take into account spherical distortions by modifying traditional image processing methods. Nevertheless, these works suffer from the following drawbacks:

- Learning-based methods. Several works [7,8,9] propose to train the network on omnidirectional datasets. However, as discussed in previous paragraphs, the existing spherical datasets are limited to indoor scenes with few images compared to the perspective benchmarks.
- Adaptation-based methods. Several works add distortion awareness over the features from the latent space by using a specific mathematical formulation, such as the fast Fourier transform [10,11] or polyhedra [12]. Despite the elegance of these solutions, the adapted network needs to be trained from scratch with specific training datasets. In addition, the adaptation methods are very demanding in terms of the computational cost. Therefore, it is difficult to implement such methods on edge devices for real-time robotic applications.

To address the abovementioned dilemmas, in this paper, we propose to directly replace standard convolution operations with distortion-aware convolutions. Therefore, we can benefit from all the development on perspective images to boost the performance on various tasks with omnidirectional images. Technically, we modify the shape of each convolution kernel according to its latitude in the image. It is worth noting that the adapted convolution has demonstrated its effectiveness in perspective networks [8,13,14,15,16,17,18]. Different from previous works [15,16,17] that dynamically learn the new kernel shape, we propose a distortion-aware convolution with our statically computed receptive field. One major advantage is that our method does not require additional training and can be directly implemented in any existing convolutional network pretrained with perspective images. The effect of spherical adaptation on optical flow estimation was proven in a previous publication [14]. Here, we extend this previous work using a state-of-the-art optical flow estimation network and generalize the demonstration to two commonly used visual modalities: semantic segmentation and monocular depth.

We compare our adapted network with its baseline version on complex and unstructured outdoor datasets. We also present a new equirectangular photorealistic forest dataset with ground-truth semantic segmentation and depth. Finally, we test our solution on real outdoor images taken with an omnidirectional camera. In all cases, the adapted networks outperform their non-spherical counterparts.

The structure of this paper is as follows. First, Section 2 presents the proposed spherical adaptation using distortion-aware convolutions. Then, a brief overview of the three visual modalities is proposed in Section 3, along with the presentation of the selected networks for the spherical adaptation. Finally, Section 4 provides the comparison results between the adapted models and their baselines on virtual and real outdoor equirectangular images.

## 2. Distortion-Aware Convolutions

The proposed spherical adaptation is based on distortion-aware convolutions. First, we present the mathematical model using a local perspective projection of the kernels on the sphere. Then, we describe the implementation and use of this adaptation on perspective networks.

### 2.1. Local Perspective Projection on the Sphere

The original adaptive convolution was initially presented by [16], where the authors proposed to learn the offsets in an end-to-end manner. More recent works exploit this idea by using fixed offsets. In [13], the authors show that the depth prior can be used to compute the adaptive kernel statically, leading to better awareness of the geometry. An adaptive convolution was also exploited in omnidirectional images [8]. The standard perspective kernel is modified to fit the equirectangular distortions. To build a kernel of resolution *r* and angular size α centered in a location $p_{00}=\left(u_{00},v_{00}\right)$ in the equirectangular image, the center coordinates are first transformed to spherical system $p_{s,00}=\left(\phi_{00},\theta_{00}\right)$ using

(1) $$\phi_{00}=\left(u_{00}-\frac{W}{2}\right)\frac{2\pi }{W}\;;\;\theta_{00}=-\left(v_{00}-\frac{H}{2}\right)\frac{\pi }{H},$$

where *W* and *H* are, respectively, the width and the height of the equirectangular image in pixels. Each point of the kernel is defined by

(2) $${\hat{p}}_{spher,ij}=\left[\begin{array}{c} {\hat{x}}_{ij}\\ {\hat{y}}_{ij}\\ {\hat{z}}_{ij} \end{array}\right]=\left[\begin{array}{c} i\\ j\\ d \end{array}\right],$$

where *i* and *j* are integers in the range $\left[-\frac{r-1}{2},\frac{r-1}{2}\right]$ and *d* is the distance from the center of the sphere to the kernel grid. In order to cover the field of view α, the distance is set to $d=\frac{r}{2\tan\left(\frac{\alpha }{2}\right)}$. The coordinates of these points are computed by normalizing and rotating them to align the kernel center on the sphere. Therefore,

(3) $$p_{spher,ij}=\left[\begin{array}{c} x_{ij}\\ y_{ij}\\ z_{ij} \end{array}\right]=R_{y}\left(\phi_{00}\right)R_{x}\left(\theta_{00}\right)\frac{{\hat{p}}_{spher,ij}}{\left|{\hat{p}}_{spher,ij}\right|},$$

where $R_{a}\left(\beta \right)$ stands for the rotation matrix of an angle β around the *a* axis. These coordinates are transformed to latitude and longitude in the spherical domain using

(4) $$\phi_{ij}=\arctan\left(\frac{x_{ij}}{z_{ij}}\right)\;;\;\theta_{ij}=\arcsin\left(y_{ij}\right);$$

and finally back-projected to the original 2D equirectangular image

(5) $$u_{ij}=\left(\frac{\phi_{ij}}{2\pi }+\frac{1}{2}\right)W\;;\;v_{ij}=\left(-\frac{\theta_{ij}}{\pi }+\frac{1}{2}\right)H.$$

**Figure 1:**
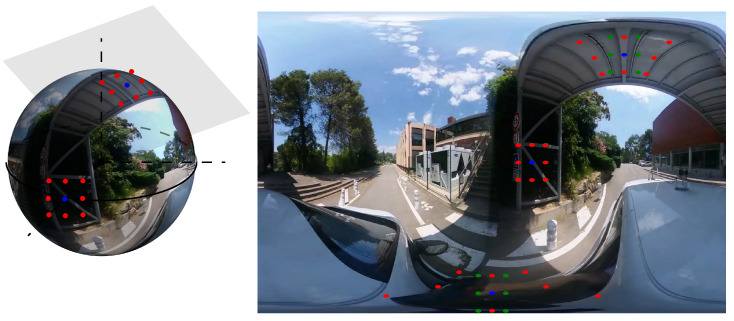
The equirectangular image presents significant distortions in the polar regions. Convolution kernel shapes are modified according to their latitude. Blue: kernel center; Green: perspective kernel; Red: adapted equirectangular kernel.

In Figure 1, some example of kernels at different latitude and longitude are presented. The blue point defines the center of the kernel $p_{00}=\left(u_{00},v_{00}\right)$. The red points are the positions of the elements $p_{ij}=\left(u_{ij},v_{ij}\right)$ in the adapted kernel, defined as previously. The green points are the positions of elements in a standard perspective kernel given by:

(6) $$u_{persp,ij}=u_{00}+ir\;;\;v_{persp,ij}=v_{00}+jr.$$

### 2.2. Implementation in a Perspective Network

**Figure 2:**
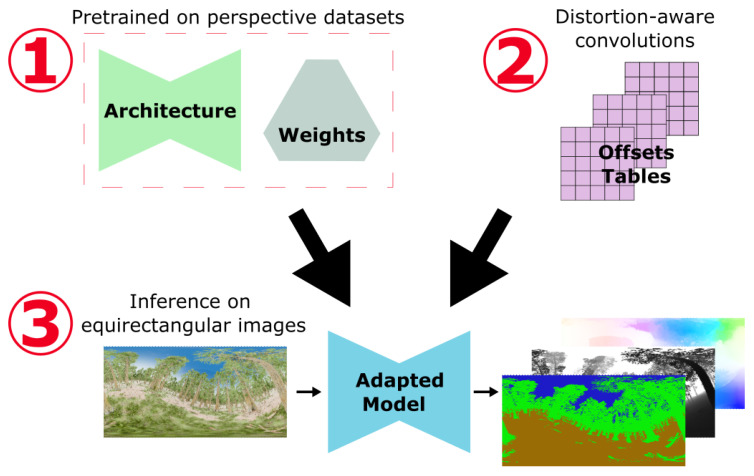
General adaptation process: 1. The architecture and weights come directly from pretraining on perspective datasets. 2. The convolution layers are modified using a precomputed shift table that takes into account equirectangular distortions. 3. Finally, we directly use spherical images as input in the adapted model to predict the modalities for which the network was pretrained.

The distortion-aware convolution strategy does not require additional parameter learning. As a result, it avoids using large and complex spherical datasets for training. Figure 2 presents a schema of the general implementation process.

The overall architecture and weights of the network are derived from a model trained in a supervised manner using perspective images and ground-truth modalities. We directly reuse the code and pretrained weights provided by the models’ authors. This highlights the simplicity of our solution integration into previously published work and ensures good performance fidelity to the original publication.

We replace the standard convolution layers with new layers handling the equirectangular distortions. In practice, the convolution operations are modified to add fixed offsets to each coordinate of the kernel points. These offsets are calculated using Equation (5), presented in Section 2.1. It only requires the input sizes and the convolution parameters. These offsets tables can be computed offline. As a result, there is no slowdown in the execution of the adapted network.

The proposed solution is compatible with every kernel, stride, or padding size. Therefore, this plugin can be implemented in any convolutional neural network architecture.

## 3. Tested Visual Modalities

Most of the latest computer vision methods are based on convolutional neural networks. To demonstrate the simplicity and versatility of our adaptation solution, we propose to implement it on several networks used for very different vision tasks. We have selected three commonly used vision tasks: semantic segmentation, depth, and optical flow. Each modality has very distinct requirements, which challenges the robustness of our solution. This section presents the three different visual modalities studied and the associated selected networks.

To highlight the generalization of our demonstration, we selected three models of very different sizes and accuracies: from the state-of-the-art heavyweight network to the ultra-lightweight architecture for resource-constrained devices.

Moreover, to demonstrate the efficiency and simplicity of our solution, we selected networks with pretrained weights provided by the models’ authors on perspective datasets. This also guarantees good performance fidelity with respect to the initial publication.

### 3.1. Semantic Segmentation

Semantic segmentation is an essential task in robotic vision. It provides a dense understanding of the different locations and object categories present in the image with pixel-level accuracy. This offers abundant cues for upper-level navigation operations. Furthermore, thanks to omnidirectional cameras, a moving agent can obtain a holistic and precise understanding of its surroundings.

To estimate the semantic segmentation in outdoor images, we choose the solution published by the MIT Scene Parsing team [19]. They propose a classical encoder–decoder architecture trained on the ADE20K dataset. This dataset contains 20,000 mixed indoor and outdoor scenes with 150 semantic classes.

The chosen architecture uses the ResNet50 dilated version as the encoder and PPM-deepsup as the decoder.

The ADE20K dataset contains 150 different classes that are sometimes semantically close. Therefore, the semantic segmentation network identifies some objects in our test dataset from the same ground-truth class as two different categories. To regroup these predictions, we combine some closely related classes. The final tree class regroups trees, plants, and canopy classes. The ground class regroups ground, earth, path, dirt, mountain, and hill classes.

### 3.2. Optical Flow

Optical flow estimation methods aim to compute the apparent motion of pixels between two frames. It enables autonomous vehicles and robots to acquire temporal cues of the surrounding scenes. In a previous publication [14], we presented a method in omnidirectional images improved by spherical adaptation. In this paper, we generalize and update that earlier work. At that time, we implemented our solution on LiteFlowNet2 [20], one of the leading algorithms in 2020. Since then, optical flow methods have been improved, mainly thanks to Transformers networks for the pixel correlation operation. However, these networks do not use convolutional layers in their core but still rely on CNNs to extract low-level features from RGB inputs before processing them. This encoding is crucial for further image processing, and we propose to adapt it to take into account distortions in equirectangular images.

We select the solution GMFlow proposed by [21], which is currently one of the leading optical flow estimation algorithms.

### 3.3. Monocular Depth

Monocular depth estimation is an important computer vision task that aims to predict dense depth maps based on a single image. Thanks to its robustness to scene changes, this is the most commonly used visual modality for obstacle avoidance in navigation.

We select the MIDAS [22] network to test our solution. This supervised model presents a classical encoder–decoder architecture, mainly built to be embedded in resource-constrained devices such as drones, resulting in one of the lightest depth estimation networks. In addition, this network is highly versatile thanks to its training on multiple indoor and outdoor perspective datasets. We specifically choose the midas_v21_small pretrained version of the network.

## 4. Results

This section compares the spherically adapted network and the baseline version. First, we provide a quantitative comparison of the virtual outdoor datasets. Then, we further investigate the differences using samples from the previously studied virtual datasets or real equirectangular images. For the latter part, we capture images of various outdoor scenes with an omnidirectional camera and analyze the differences.

### 4.1. Testing Datasets

Outdoor scenes are generally more challenging for networks than indoor scenes, mainly due to the diversity of lighting and the limited amount of outdoor images in the training datasets. However, the available outdoor omnidirectional datasets are very limited and do not include multiple visual modalities ground truths. In addition, forest images are often not used in perspective training datasets which further tests the robustness of the tested models. Therefore, forest scenes are an ideal environment to challenge the networks presented above.

For semantic segmentation and monocular depth estimation, we build a photorealistic forest environment RWFOREST. Unfortunately, the ground-truth extraction of the spherical optical flow is not yet available in this environment. As a result, we use two other datasets to test this visual modality: OmniFlowNet [14] and Flow360 [23]. Table 1 summarizes the different equirectangular datasets used to test the various adapted networks. A more detailed presentation of the different environments is provided in the sections below.

#### 4.1.1. RWFOREST Dataset

**Figure 3:**
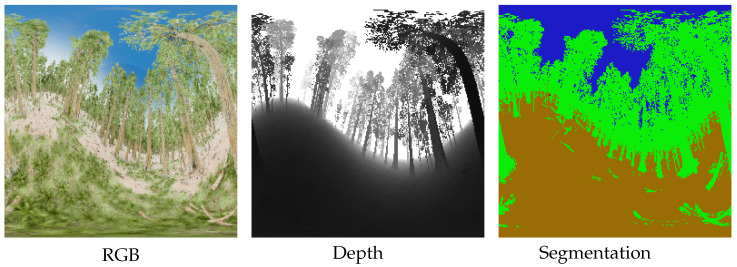
RWFOREST 256 × 256 dataset.

Using the best rendering capabilities of Unreal Engine [24] and the forest textures from its marketplace, we create a photorealistic forest environment with complex lighting and dense foliage. We propose, in this paper, RWFOREST: a dataset of 1000 equirectangular RGB images with associated ground-truth depth and semantic segmentation provided by the AIRSIM [25] plugin. Three semantic classes are distinguished: trees, ground, and sky. The image resolution is 256×256 for all. Additional results on higher resolutions are provided in Appendix A. Figure 3 presents a sample of the RWFOREST dataset.

#### 4.1.2. OmniFlowNet and Flow360 Datasets

The OmniFlowNet dataset, published in [14], features three different scenes called CartoonTree, Forest, and LowPolyModel. These sets are generated using Blender [26], with free 3D models available online. This dataset gathers 1200 equirectangular RGB images with an associated ground-truth optical flow. The Flow360 dataset, published by [23], proposes several urban driving scenes during different times of the day or weather. This dataset provides the ground-truth omnidirectional optical flow associated with RGB image sequences.

**Figure 4:**
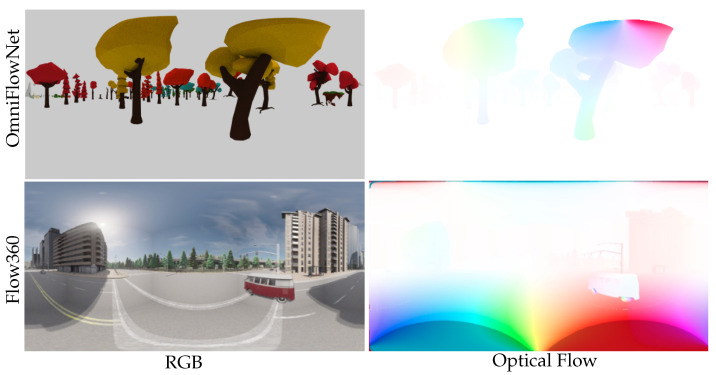
OmniFlowNet [14] and Flow360 [23] datasets. Equirectangular ground-truth optical flow is associated to the RGB images sequence.

Figure 4 shows a brief overview of the OmniFlowNet and Flow360 datasets.

### 4.2. Quantitative Comparison on Virtual Outdoor Datasets

To facilitate the reading of the results, we have grouped in Table 2 the comparison of the different visual modalities. To do so, we selected an error metric specific to each modality: we use the complement of the Mean Intersection Over Union (1−MIoU) for the semantic segmentation, the End-Point Error for the optical flow, and the Relative Absolute Error for the monocular depth. We also offer additional comparison metrics in Appendix A. In addition, the definitions of all the metrics used are provided in Appendix B.

The metric comparison reveals that adapted networks with distortion-aware convolutions always perform better than their counterparts from the baseline perspective. By observing this persistent improvement for all the modalities considered, we conclude that our proposed adaptation approach has excellent generalization capabilities. The improvement is consistent despite very different modality needs, network architectures, and training datasets. Moreover, the convolution operation modification appears robust. The gain in the error metric exceeds 3% on each modality except on the Flow360 dataset.

The lack of periodicity in the estimated optical flow can explain this Flow360 smaller gain. The spherical optical flow is periodic, but the network did not learn this information when learning the perspective. Thus, the estimation of the road pixel flow is still inaccurate. This lack of periodicity remains one of the limitations of this adaptation method for optical flow networks. However, modified convolutions still improve the predictions, especially in the case of single-object flow prediction, as shown in the following qualitative study.

We provide below a qualitative analysis to further explore the differences in the prediction between all the models considered.

### 4.3. Qualitative Comparison on Real and Virtual Outdoor Datasets

This qualitative comparison presents sample predictions of the three modalities studied on the proposed virtual outdoor datasets. We also compare real outdoor images taken with a RICOH THETA Z1. We looked for specific activations in the polar regions of the equirectangular images during the scene creation.

#### 4.3.1. Semantic Segmentation

**Figure 5:**
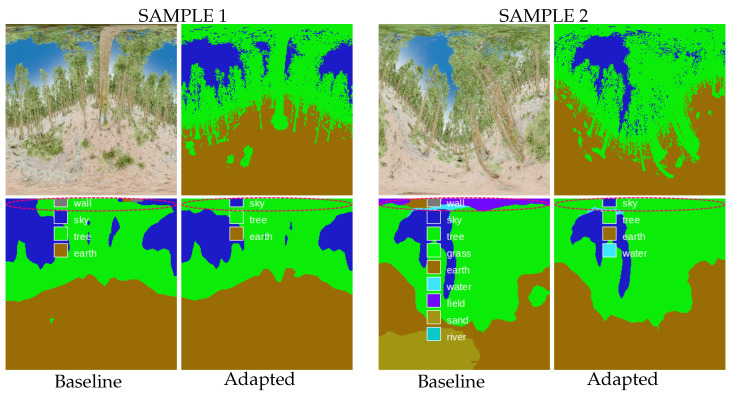
Prediction examples in the RWFOREST dataset. The spherical adaptation improves shape detection (tree canopy is better identified) and reduces erroneous class estimation. (**Top left**): RGB input, (**top right**): ground-truth segmentation, (**bottom left**): prediction from the baseline network, (**bottom right**): prediction from the adapted network.

This section compares the semantic segmentation estimation differences between the adapted and baseline networks. Looking at the set of predictions made on the RWFOREST dataset, we notice two main improvements: better detection of the shapes in the polar regions and a less erroneous class estimation. Figure 5 shows the two prediction samples used to illustrate these results.

First, the spherical adaptation helps the network to take into account equirectangular distortions. The detection of shapes and objects is improved in highly distorted regions thanks to a better local coherence of the pixels. This effect is visible in sample 1, where the adapted network better identifies the tree canopy (upper polar region of the image).

In addition, the adaptation also reduces the number of noisy predictions. Some objects in the equirectangular images are highly distorted, resulting in false class predictions by the network. In sample 2, the upper polar region of the adapted version is less noisy and contains almost no false predictions.

**Figure 6:**
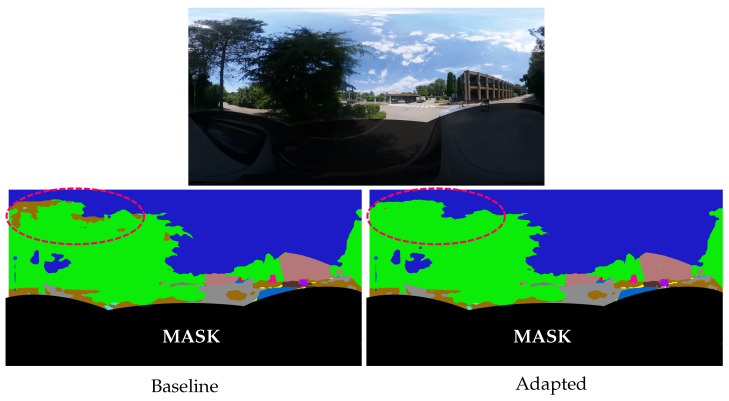
Urban driving example. The adapted network better identifies the tree canopy. A red circle at the top left of the image highlights the area with the most visible differences: the baseline network estimates the earth (in brown) class instead of trees (in green).

We observe the same findings when estimating the semantic segmentation in real urban driving scenes. In our proposed example, Figure 6, we captured images when the car was passing under trees in order to focus on the tree canopy detection. The semantic segmentation predicted by the adapted network is more accurate than the baseline estimate, with better tree canopy identification and less noisy class predictions. This confirms that distortion-aware convolutions improve the semantic segmentation in virtual and real outdoor images.

A mask is added to the image’s lower part to hide the car’s semantic segmentation estimate. Indeed, the car’s shape is strongly distorted due to its proximity to the omnidirectional camera. The absence of such images and nearby objects in the training dataset makes the network unable to make a correct prediction. Spherical adaptation improves the quality of semantic segmentation in spherical images but remains limited by the training dataset, as in all supervised methods.

#### 4.3.2. Monocular Depth

Monocular depth prediction is more difficult to comment on than semantic segmentation because depth differences are less visible to the human eye. The visual results seem ambiguous, and it is challenging to decide which estimate is better than the other. Therefore, a more detailed quantitative comparison is provided in Table 3. Additional metrics are provided, all of which show that the depth prediction from the spherical adapted network is more accurate than the baseline version.

**Figure A1:**
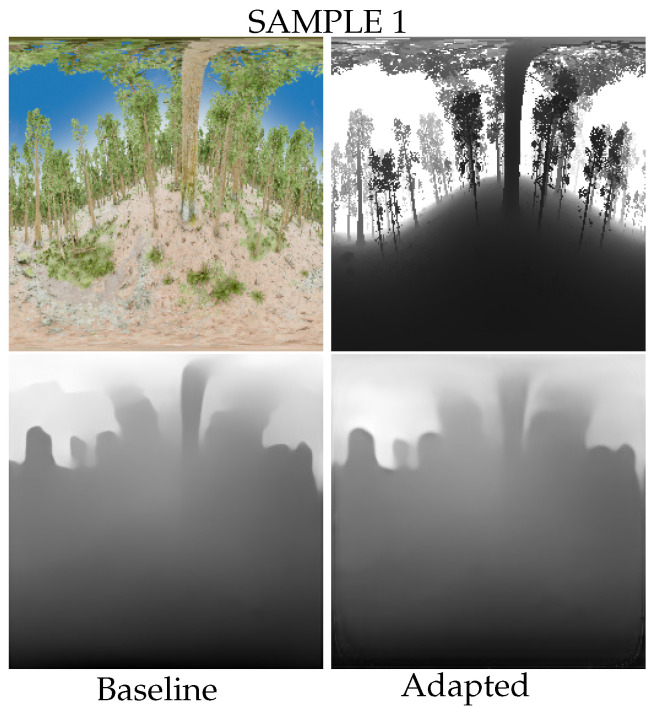
Prediction examples in the RWFOREST dataset. The predicted depth images are visually challenging to compare. However, quantitative measurements have shown that the adapted version is numerically better than the baseline. Top left: RGB input, top right: ground-truth monocular depth, bottom left: prediction from the baseline network, bottom right: prediction from the adapted network.

In the appendix, Figure A1 shows the monocular depth estimation from the same RGB images used in the semantic segmentation prediction example.

**Figure 7:**
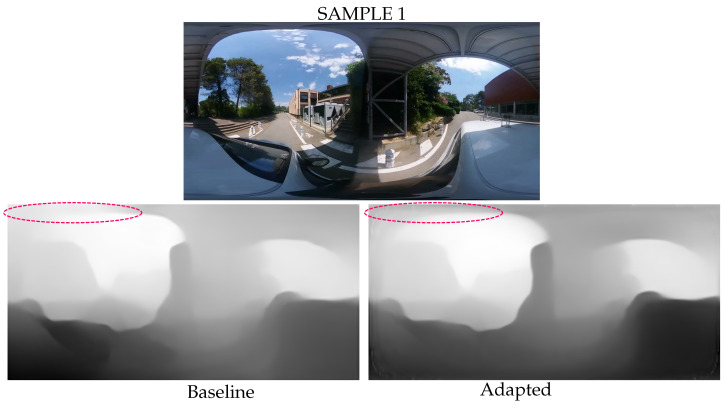
Urban driving examples. The adapted network better estimated depth in the polar regions of the equirectangular images. A red circle at the top left of the image highlights the area with the most visible differences.

**Figure 8:**
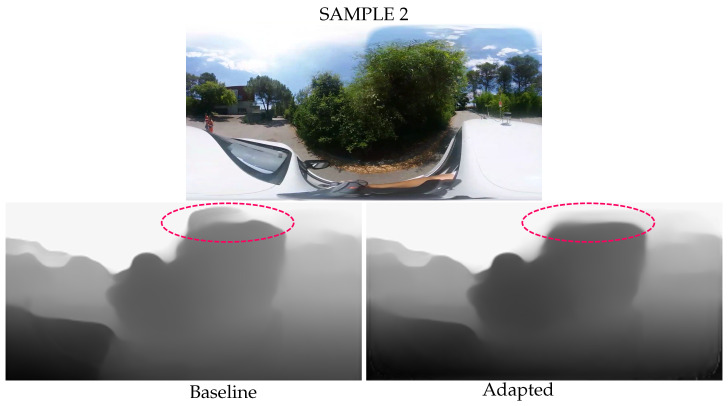
Less erroneous depth estimation from the adapted network. A red circle at the top of the image highlights the area with the most visible differences.

For real image examples, we focus on predicting the distance of objects in the upper polar region during urban driving scenes. Figure 7 and Figure 8 show two image acquisitions, the first as the car passes under a bridge and the second as it drives by a large tree. Similarly to the results on the virtual images and semantic segmentation examples, the detection of shapes is improved in the polar regions and there is less erroneous depth estimation. Sample 1 shows that the spherical adaptation improves the depth prediction in the polar regions of the equirectangular images. In the upper left of the image, the bridge depth estimation is more accurate and smoother due to better local pixel coherence. In addition, sample 2 shows that the adapted prediction is less sensitive to illumination noise. The image contrast in the top polar region shows significant differences due to the sun configuration. The baseline network interprets these changes as depth differences, while the adapted model is more robust and remains accurate.

#### 4.3.3. Optical Flow

**Figure 9:**
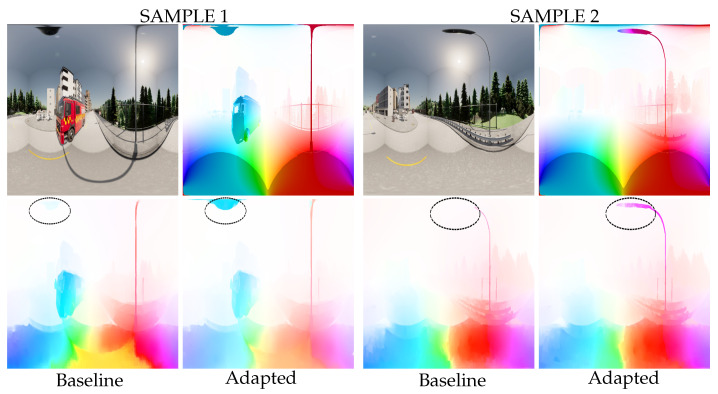
Prediction examples in the Flow360 dataset. Spherical adaptation allows better tracking of objects moving in polar regions. As a result, the estimation of the optical flow of the observed lamp post is significantly improved (area highlighted by the red circle). (**Top left**): RGB input, (**top right**): ground-truth optical flow, (**bottom left**): prediction from the baseline network, (**bottom right**): prediction from the adapted network.

The optical flow enhancements are clearly visible as objects move into the polar regions of the equirectangular image. Figure 9 shows two optical flow estimates in the dataset Flow360. In both examples, the car passes under a streetlight. Due to the improved local pixel coherence provided by the distortion-aware convolutions, the adapted network is able to track the path of the streetlight in the upper polar region of the image. As a result, the estimated optical flow is close to the ground truth. In parallel, the non-adapted network has difficulty detecting this same streetlight. Consequently, the flow prediction is inaccurate in sample 1 or even empty in sample 2.

**Figure 10:**
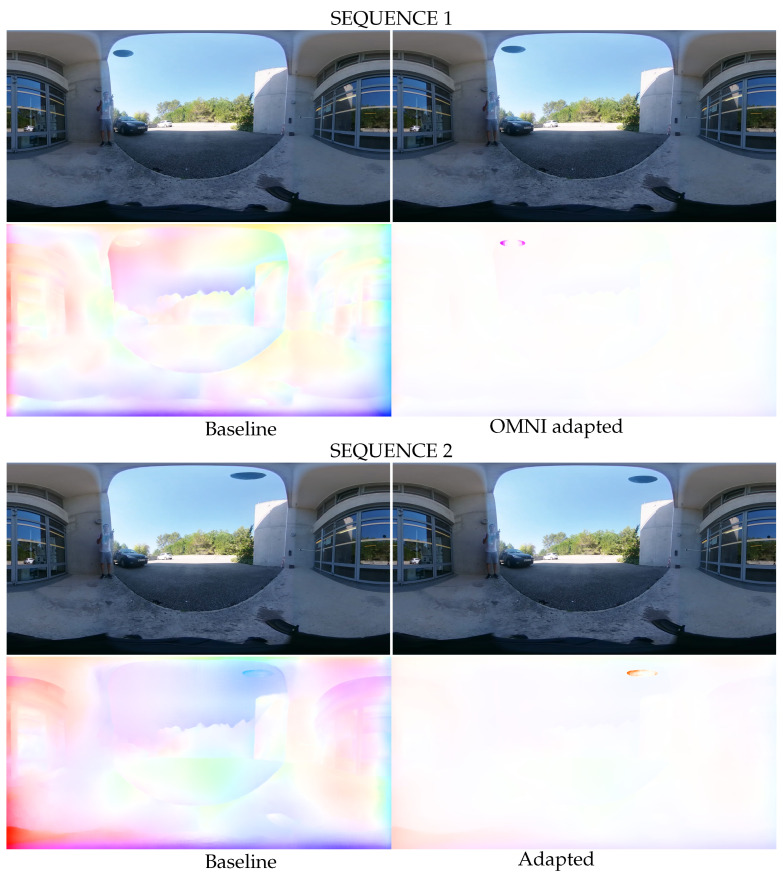
Ball throw example. The adapted network provides correct optical flow estimation, whereas the baseline version loses track of the ball. Top left: RGB input frame at *t*, top right: RGB input frame at t+1, bottom left: prediction from the baseline network, bottom right: prediction from the adapted network.

For optical flow estimation in real images, we focus on the motion of a ball during a throw. Figure 10 shows two different image sequences with associated optical flow predictions. Due to better local pixel coherence, the adapted model keeps track of the ball and provides an accurate motion estimate. In contrast, the baseline network loses track of the ball, resulting in a noisy optical flow prediction without an apparent precise motion. This result confirms the improvement in the optical flow estimation in virtual and real images provided by distortion-aware convolutions.

## 5. Conclusions

This paper presents a generalization of the spherical adaptation of perspective methods to equirectangular images using distortion-aware convolutions. We have tested and proved the adaptation of three fundamental visual modalities in computer vision: semantic segmentation, optical flow, and monocular depth.

A state-of-the-art network was modified for each modality to take into account the spherical distortions with a simple and fast adaptation without architecture modification or additional training. When tested on virtual equirectangular outdoor images, the adapted version outperformed its baseline in all cases. Furthermore, regardless of the visual modality, the network estimations were improved in highly distorted regions. The predictions were smoother thanks to better local pixel coherence. Furthermore, there were less erroneous estimations. We observed the same results when applying these methods to real outdoor equirectangular images.

Therefore, although this solution does not compete with networks specializing in spherical images, it allows the simple and fast adaptation of any architecture. Furthermore, this can easily overcome the lack of outdoor omnidirectional datasets. Finally, it allows us to keep up with the new architectures proposed regularly in deep learning for perspective images.

## Figures and Tables

**Table 1:** Equirectangular datasets used to test the different adapted networks. The visual ground-truth modalities present in the dataset are pinpointed, always associated with equirectangular RGB images.

	Dataset	RWFOREST 256 × 256	OmniFlowNet [14]	Flow360 [23]
Visual Modality				
Semantic Segmentation		√	×	×
Monocular depth		√	×	×
Optical flow		×	√	√
Number of images		1000	1200	1400
Resolution		256×256	768×384	1024×512

**Table 2:** Comparison of adapted and baseline networks on three different visual modalities. The error metric used for semantic segmentation is the complement of the Mean Average of Intersection Over Union, for optical flow is the End-Point Error, and for depth is the Absolute Relative Error.

	Error Metric (↓)
Semantic segmentation baseline ${}^{1}$	0.323
Semantic segmentation adapted ${}^{1}$	**0.312 (−3.4%)**
Monocular depth baseline ${}^{1}$	1.198
Monocular depth adapted ${}^{1}$	**1.154 (−3.673%)**
Optical flow baseline ${}^{2}$	5.16
Optical flow adapted ${}^{2}$	**4.96 (−3.93%)**
Optical flow baseline ${}^{3}$	16.15
Optical flow adapted ${}^{3}$	**15.95 (−1.27%)**

For each comparison, the best results are in **bold**. ^1^ Evaluated on RWFOREST 256 × 256. ^2^ Evaluated on OmniFlowNet. ^3^ Evaluated on Flow360.

**Table 3:** Comparison of adapted and baseline depth estimation networks on RWFOREST dataset. The definition of every metric is provided in Appendix B.

RWFOREST 256 × 256	δ<1.25 (↑)	$\delta <1.25^{2}$ (↑)	$\delta <1.25^{3}$ (↑)	AbsRel (↓)	SqRel (↓)	RMSE (↓)
Monocular depth baseline	0.251	0.440	0.619	1.198	0.451	0.277
Monocular depth adapted	**0.26 (+3.586%)**	**0.454 (+3.182%)**	**0.630 (+1.777%)**	**1.154 (−3.673%)**	**0.443 (−1.774%)**	**0.275 (−0.722%)**

For each comparison, the best results are in **bold**.

## Data Availability

Our code implementation will be available on GitHub at https://github.com/COATZ/OMNI-CONV.

## Appendix A. Additional Evaluation Metrics

To compare the adapted and baseline version of each model, we used comparison metrics specific to each visual modality estimated. The additional results are presented in this section. The definition of every metric is provided in Appendix B.

### Appendix A.1. Semantic Segmentation Evaluation

**Table A1:** Comparison of adapted and baseline semantic segmentation networks on RWFOREST 256 × 256, RWFOREST 512 × 256, and RWFOREST 1024 × 512.

RWFOREST 256 × 256	MIoU (↑)	Accuracy (↑)	AECE (↓)
Semantic segmentation baseline	0.677	0.810	2.045
Semantic segmentation adapted	**0.688 (+1.525%)**	**0.828 (+2.282%)**	**0.337**
**RWFOREST 512 × 256**	MIoU (↑)	Accuracy (↑)	AECE (↓)
Semantic segmentation baseline	0.504	0.631	3.692
Semantic segmentation adapted	**0.564 (+11.980%)**	**0.639 (+1.332%)**	**1.577**
**RWFOREST 1024 × 512**	MIoU (↑)	Accuracy (↑)	AECE (↓)
Semantic segmentation baseline	0.443	0.621	5.845
Semantic segmentation adapted	**0.528 (+19.174%)**	**0.627 (+0.852%)**	**3.034**

For each comparison, the best results are in **bold**.

### Appendix A.2. Monocular Depth Evaluation

**Table A2:** Comparison of adapted and baseline depth estimation networks on RWFOREST 256 × 256, RWFOREST 512 × 256, and RWFOREST 1024 × 512.

RWFOREST 256 × 256	δ<1.25 (↑)	$\delta <1.25^{2}$ (↑)	$\delta <1.25^{3}$ (↑)	AbsRel (↓)	SqRel (↓)	RMSE (↓)
Monocular depth baseline	0.251	0.440	0.619	1.198	0.451	0.277
Monocular depth adapted	**0.26 (+3.586%)**	**0.454 (+3.182%)**	**0.630 (+1.777%)**	**1.154 (−3.673%)**	**0.443 (−1.774%)**	**0.275 (−0.722%)**
**RWFOREST 512 × 256**	δ<1.25 (↑)	$\delta <1.25^{2}$ (↑)	$\delta <1.25^{3}$ (↑)	AbsRel (↓)	SqRel (↓)	RMSE (↓)
Monocular depth baseline	0.254	0.421	0.554	1.664	0.739	0.305
Monocular depth adapted	**0.264 (+3.937%)**	**0.437 (+3.8%)**	**0.574 (+3.610%)**	**1.567 (−5.829%)**	**0.695 (−5.954%)**	**0.298 (−2.295%)**
**RWFOREST 1024 × 512**	δ<1.25 (↑)	$\delta <1.25^{2}$ (↑)	$\delta <1.25^{3}$ (↑)	AbsRel (↓)	SqRel (↓)	RMSE (↓)
Monocular depth baseline	0.248	0.413	0.545	1.836	0.856	0.316
Monocular depth adapted	**0.257 (+3.629%)**	**0.431 (+4.358%)**	**0.57 (+4.587%)**	**1.707 (−7.026%)**	**0.79 (−7.70%)**	**0.307 (−2.848%)**

For each comparison, the best results are in **bold**.

### Appendix A.3. Optical Flow Evaluation

**Table A3:** Comparison of adapted and baseline optical flow networks on OmniFlowNet, Flow360, CityScene, and EquirectFlyingThings datasets.

	OmniFlowNet Dataset [14]	Flow360 Dataset [23]
	EPE (↓)	EPE (↓)
Optical flow baseline	5.16	16.15
Optical flow adapted	**4.96 (−3.93%)**	**15.95 (−1.27%)**
	**CityScene** [9]	**EquirectFlyingThings** [9]
	EPE (↓)	EPE (↓)
Optical flow baseline	32.16	42.44
Optical flow adapted	**31.36 (−2.08%)**	**41.83 (−1.43%)**

For each comparison, the best results are in **bold**.

## Appendix B. Estimation Metrics

### Appendix B.1. Semantic Segmentation Metrics

In order to compare the performances of the spherical adapted network version and its baseline, we use two common semantic segmentation metrics [27]:

- The Mean Intersection Over Union (MIoU), which indicates the intersection over union between predicted and ground-truth pixels, averaged over all the classes;
- The Mean Accuracy, which indicates the proportion of correctly classified pixels averaged over all the classes;
- The Averaged Erroneous Class Estimate (AECE), which indicates the number of classes detected by the network but not present in the ground-truth image averaged over all runs.

### Appendix B.2. Monocular Depth Metrics

To compare the baseline and modified networks, we use three commonly used metrics in depth prediction [28]:

- Accuracy under a threshold th:
  (A1) $$\delta =max(\frac{\hat{d_{p}}}{d_{p}},\frac{d_{p}}{\hat{d_{p}}})<th;$$
- Absolute Relative Error:
  (A2) $$AbsRel=\frac{1}{T}\sum_{p}\frac{|d_{p}-\hat{d_{p}}|}{d_{p}};$$
- Linear Root Mean Square Error:
  (A3) $$RMSE=\sqrt{\frac{1}{T}\sum_{p}{\left(d_{p}-\hat{d_{p}}\right)}^{2}};$$
  with $\hat{d_{p}}$ the estimated depth for a pixel *p*, $d_{p}$ the ground-truth depth, and *T* the total number of pixels.

### Appendix B.3. Optical Flow Metric

In order to compare the performances of the spherical adapted network version and its baseline, we use the End-Point Error EPE [29]. If the flow vector estimated is $\left(u_{f},v_{f}\right)$ and the ground-truth flow $\left(u_{fgt},v_{fgt}\right)$, then the resulting metric is:

(A4) $$EE=\frac{1}{N}\sum_{N}^{}\sqrt{{\left(u_{fgt}-u_{f}\right)}^{2}+{\left(v_{fgt}-v_{f}\right)}^{2}}.$$
